# Molecular phylogeny, ecology and multispecies aggregation behaviour of bombardier beetles in Arizona

**DOI:** 10.1371/journal.pone.0205192

**Published:** 2018-10-31

**Authors:** Jason C. Schaller, Goggy Davidowitz, Daniel R. Papaj, Robert L. Smith, Yves Carrière, Wendy Moore

**Affiliations:** 1 Department of Entomology, University of Arizona, Tucson, Arizona, United States of America; 2 Department of Ecology and Evolutionary Biology, University of Arizona, Tucson, Arizona, United States of America; University of Arkansas, UNITED STATES

## Abstract

Aggregations of conspecific animals are common and have been documented in most phyla. Multispecies aggregations are less common and less well studied. Eight species of *Brachinus* beetles —famous for their unique, highly effective, chemical defense—regularly settle together form large diurnal multispecies aggregations in dark, moist areas in riparian habitats in the Sonoran Desert Region. Here, we document these multispecies aggregations and investigate the incidence and dynamics of aggregation behavior. Analysis of species composition of 59 field-collected aggregations revealed that 71% contained more than one species, eight species regularly co-occurred in aggregations, and no two species showed a preference to aggregate with one another. We provide the first phylogenetic analyses of participants in multispecies aggregations, and find that *Brachinus* species found together in aggregations are not each other’s closest relatives but rather are dispersed throughout the phylogeny of the genus. Further, we find no tendency for species to aggregate with close relatives more frequently than distant relatives. Laboratory experiments on *B*. *elongatulus* showed that it chose to settle in occupied shelters over empty shelters. Experiments with *B*. *hirsutus* and *B*. *elongatulus* showed that *B*. *hirsutus* prefers to settle under shelters housing heterospecifics over conspecifics. Our findings suggest that these multispecies aggregations do not form by chance, but rather are initiated by a genus-wide aggregation cue associated with the presence of individuals already in a shelter, which is likely to be chemical and potentially tactile in nature.

## Introduction

Aggregations of conspecifics in the context of foraging, mating, or breeding are common in the animal kingdom. Multispecies aggregations are less common but occur across a wide array of metazoan taxa and serve functions including deterring predators, promoting cooperative foraging, and facilitating mating [1, 2]. In birds, for example, flocks and nesting sites may comprise several species [3, 4]. Yellow warblers nest in aggregations that include larger and more aggressive species such as red-winged blackbirds and grey catbirds, which reduces the risk of brood parasitism of yellow warblers from brown-headed cowbirds [3]. While temporary multispecies associations are well documented among arthropods that specialize on, and that are attracted to, ephemeral food resources and/or oviposition sites, such as carrion, rotting cacti, dung and fungi, species known to regularly form multispecies aggregations for resting are extremely rare among arthropods with only a few cases being reported in harvestmen (Arachnida: Opiliones) [5], net-winged beetles (Coleoptera: Lycidae) [6–7], whirligig beetles (Coleoptera: Gyrinidae) [8] and ground beetles (Coleoptera: Carabidae) [9–11]. Interestingly all documented examples of multispecies aggregations among arthropods that form by individuals being attracted to one another, rather being attracted to an ephemeral food resource or oviposition site, involve species that are chemically defended. It could be that defensive chemicals play a role in both the mechanism of multispecies aggregation formation (by providing an aggregation cue) and the functional significance of these aggregations (by providing the participants greater defense).

Individuals of several carabid genera regularly settle together to form multispecies aggregations in France (Jeannel, 1942). These aggregations often include three species of the bombardier beetle genus, *Brachinus* Weber (Jeannel, 1942; Wautier, 1971). When multiple closely related species regularly settle together and form multispecies aggregations, it is interesting from both ecological and evolutionary perspectives. The very existence of multispecies aggregations seems to break basic biological principles and thus begs for explanation. For example, classical concepts of resource partitioning predict that species will not coexist if they feed on the same resource and they are not capable of differentiating their niches sufficiently in space or time [10]. Further, multispecies aggregations among close relatives might be thought to incur an increased risk of hybridization. In such cases, what maintains species boundaries?

**Fig 1:**
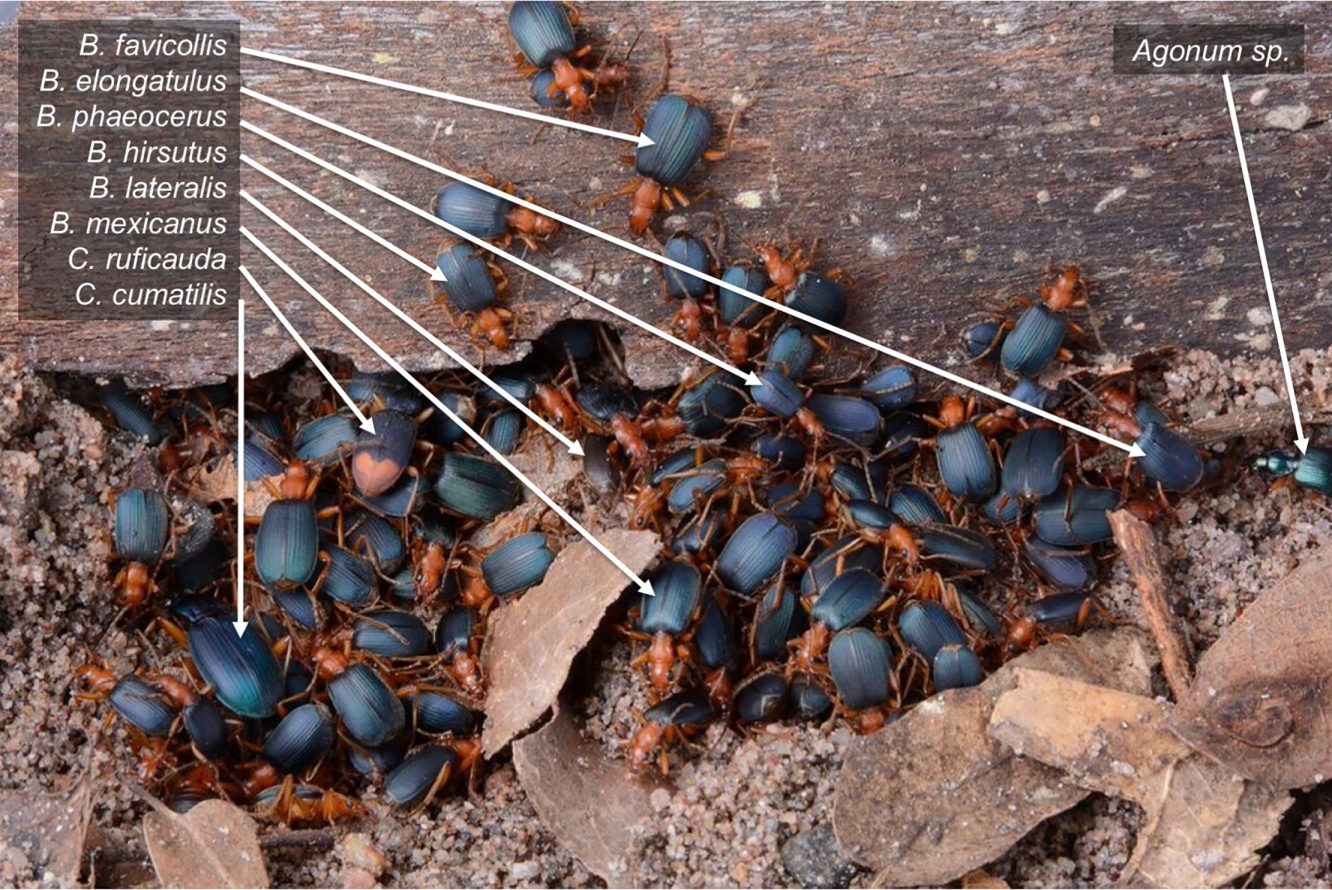
Multispecies aggregation of carabid beetles. This multispecies aggregation was photographed in the laboratory after lifting a piece of wood under which the beetles had settled. Individuals were collected along the San Pedro River and are members of the following species: *Brachinus favicollis*, *B*. *elongatulus*, *B*. *phaeocerus*, *B*. *hirsutus*, *B*. *lateralis*, *B*. *mexicanus*, *Chlaenius ruficauda*, *C*. *cumatilis*, and *Agonum sp*.

**Fig 2:**
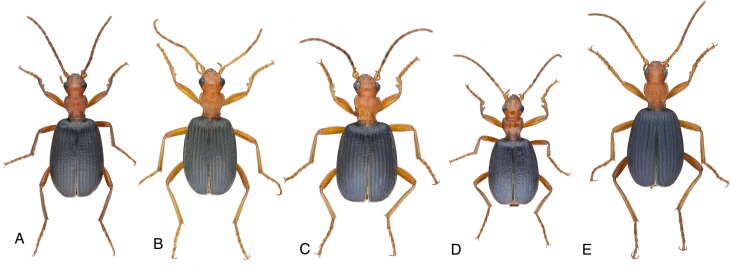
Dorsal habitus of five *Brachinus* species. Dorsal habitus of five of the eight species of *Brachinus* that form multispecies aggregations in southeastern Arizona. (a) *B*. *elongatulus*, (b) *B*. *hirsutus*, (c) *B*. *mexicanus*, (d) *B*. *phaerocerus*, (e) *B*. *favicollis*. Scale bar = 1 cm.

Here, we document and investigate a fascinating new example of multispecies aggregations involving eight species of bombardier beetle genus *Brachinus* that regularly settle together under shelters to rest throughout the day in riparian areas within the Sonoran Desert Region of Arizona (JCS, WM, RLS pers. obs.) (Figs 1 and 2). At night individuals roam alone as they actively forage for protein (especially drowned arthropods) at the water’s edge. When a large resource, such as a dead frog or toad is found, many (occasionally > 100) *Brachinus* individuals of several species may accumulate and feed together. Although they feed together in these situations we never observed individuals directly interacting at night except to mate during mating season (which varies among species). Several hours before sunrise individuals begin searching for a refuge where they will remain through the daylight hours. Suitable refuges are cavities under rocks, branches or coarse accumulations of debris underlain by moist (never dry) soil/sand that is well drained and contains no free-standing water. Potential refuges are usually abundant in any one habitat but only a small fraction of them are occupied on any one day. Over 30 years of observation, we never found a refuge occupied (and anthropogenically disturbed) on one day to be reoccupied on the next day (RLS, pers. obs.). This observation stands in contrast to reports from the aggregations in France, where the same shelter was repeatedly used and remained attractive for many years [11].

While aggregation sizes vary, virtually all aggregations contain multiple *Brachinus* species, and sometimes other riparian ground beetles including *Chlaenius* Bonelli, *Agonum* Linneaus, *Platynus* Bonelli, *Panageus* Latreille, and *Galerita* Fabricius (Fig 1). These multispecies aggregations are Müllerian associations, as the species display similar aposematic coloration. Both sexes of all species are defended against predators by chemicals produced in a pygidial defensive gland system that is homologous across all species of the beetle suborder Adephaga. Of these species, the aposematic pattern of *Brachinus* spp. is particularly consistent (Fig 2) and their defensive chemistry is particularly powerful. They explosively discharge toxic benzoquinones at temperatures reaching 100^o^ C, which they aim precisely at their predators, earning them the common name “bombardier beetles” [12–16]. The powerful defensive chemistry of bombardier beetles is effective against both invertebrate and vertebrate predators, even after beetles have been swallowed [17].

All *Brachinus* species are of similar size (Fig 1) and we assume all of the species are equally defensive. Apparently, selection has strongly favored retention of “the *Brachinus* habitus” through many speciation events, and all North American species of *Brachinus* are “look-alikes.” Traits that distinguish many of the species are not obvious and include a darkening (infuscation) of certain sclerites, setation patterns, and slight differences in the shape of the male endophallus [18]. Since reliable recognition of species is key to exploring any aspect of multispecies aggregation in this system, we constructed the first molecular phylogeny for the genus *Brachinus* to confirm our species identifications. To our knowledge this is the first phylogenetic analysis of participants in multispecies aggregations. With the phylogenetic information in hand, we additionally assessed the phylogenetic relatedness of aggregation members to determine if they are close relatives of one another or if they are more distantly related within the phylogeny of the genus.

We documented the occurrence and prevalence of multispecies aggregations from numerous localities in central and southern Arizona by collecting entire aggregations in the field and counting and identifying all individuals to the species level. Here, we address whether or not individuals of various species aggregate together in frequencies different from expected based on the relative species abundance for several locations. In the laboratory, we video-recorded individuals transitioning between foraging at night and sheltering in the morning to investigate the timing and dynamics of aggregation formation. To investigate their behavior further, we conducted choice experiments in the laboratory to test our hypothesis that multispecies aggregations are actively formed. Alternatively, multispecies aggregations might randomly form simply due to limited availability of suitable shelters and/or beetles might have a clumped distribution during the night that increases the likelihood of multiple individuals sharing a shelter during the day.

## Materials and methods

### Molecular phylogenetic analysis

Eighty-one specimens representing 36 species of *Brachinus* and 13 outgroup species were included in the analysis. Our taxon sampling included 24 specimens from the eight putative species that were collected in multispecies aggregations in Arizona, as well as specimens of other species from throughout North America, South America, Africa and Europe (see S5 Table).

Total genomic DNA was extracted from the right middle or hind leg of each voucher specimen using the Qiagen® DNeasy Blood and Tissue Kit (Qiagen, Valencia, CA), according to manufacturer suggested protocol. The barcoding region of the mitochondrial gene cytochrome oxidase subunit 1 (COI) was PCR amplified with primers LCO1490 and HCO2198 [19]. A 750 base pair fragment of the single copy nuclear gene carbamoyl-phosphate synthase 2 (CAD 2) was PCR amplified, using primers cd338F (5’ ATGAARTAYGGYAATCGTGGHCAYAA 3’) and cd688R [20]. About half of the CAD samples required a nested PCR with primers cd398F (5’ GARCAYACAGCNGGNCCNCAAGA 3’) and cd696R (5’ AANGGRTCNACRTTTTCCATATT 3’) to produce ample PCR product for sequencing. PCR products were cleaned, quantified, normalized and sequenced in both directions at the University of Arizona’s Genomic and Technology Core Facility using a 3730 or 3730XL Applied Biosystems automatic sequencer. Chromatograms were assembled and initial base calls were made for each gene with Phred [21] and Phrap [22] as orchestrated by Mesquite [23] in combination with the Chromaseq [24]. Final base calls were made after manual inspection of individual sequences in Chromaseq; universal ambiguity, IUPAC, codes were used when multiple peaks were present at individual sites. Resulting sequences were deposited in GenBank (S1 Table).

Sequences were aligned using MAFFT v 7.310 [25] with default settings. The best partitioning scheme and best fitting model of evolution were selected using PartitionFinder version 1.01 [26] based on the Akaike information criterion (AIC) scores. PartitionFinder analyses favored the following four-part partitioning scheme: codon position 1 of both genes were combined, codon position 2 of both genes were combined, and the third codon position for each gene were placed in separate partitions. Each partition was analyzed with the GTR+gamma model of evolution. Searches for optimal trees were conducted using the CIPRES Science Gateway portal [27]. Maximum likelihood (ML) heuristic searches were conducted using RAxML v8.2.8 [28]. ML searches based on the concatenated matrix included 500 alternative runs. Non-parametric bootstrap analyses were performed separately from the ML tree searches. Clade support was conducted using rapid bootstrapping with a subsequent ML search and letting RAxML halt bootstrapping automatically (using MRE-based bootstopping criterion).

### Field collections

**Fig 3:**
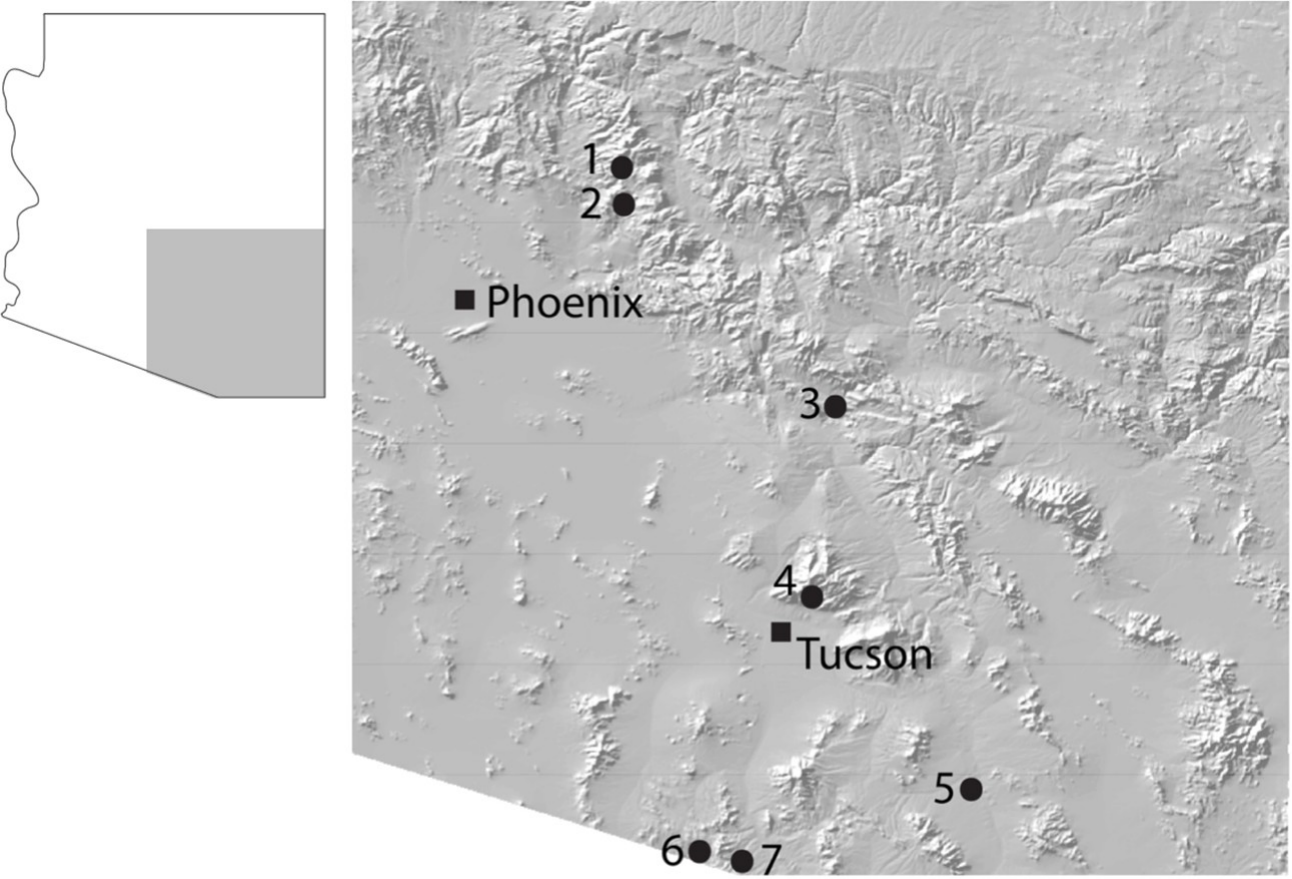
Collecting locations. Map of seven sites across central and southeast Arizona where whole aggregations of *Brachinus* were collected. For more details on each site see Table 1.

To assess the prevalence and species composition of multispecies aggregations, whole aggregations were collected from seven different localities across southeastern and central Arizona (Fig 3, Table 1). Several factors were taken into consideration in choosing these sites, including accessibility while maximizing geographical expanse. Suitable habitat of moist sand or silt along permanent or nearly permanent still, or slow moving fresh water is rather limited in this region, and extensive exploration was necessary to find these sites. All sites were in riparian habitats surrounded by Sonoran desertscrub, semi-arid grassland, juniper-pinyon woodland, or Madrean oak woodland, and all were on National Forest land, where no specific permissions are required for collecting insects by hand. Aggregations were located during the day by lifting rocks and debris in moist soil near the water’s edge. Once an aggregation was uncovered, the beetles were collected by hand, placed in a Whirl-pack ™ filled with 100% ethanol, and labeled with an aggregation number. All individuals from each aggregation were pinned, labeled, identified to species, and deposited in the University of Arizona Insect Collection (UAIC).

**Table 1:** Collection data and site descriptions.

	Collection Data	Site Description	Aggregations (Individuals)
**Site 1**	USA: AZ: Maricopa Co. Upper Sycamore Creek, off Hwy. 87 33.8991N, 111.4843W, 1098m 28 March 2011, J. Schaller	Rocky perennial creek with extensive vegetation, sycamores, cottonwoods, oaks, junipers, surrounding hills with juniper/pinyon woodland	2 (8)
**Site 2**	USA: AZ: Maricopa Co. Lower Sycamore Creek, off Hwy. 87 33.7963N, 111.4938W, 722m 28 March 2011, J. Schaller	Wide silty wash with intermittent pools and rocky areas, few sycamores, mesquites, surrounding hills with Sonoran desertscrub	16 (182)
**Site 3**	USA: AZ: Pinal Co. 3 mi NE Winkleman, The Shores Rec. Area Gila Riv. 33.0211N, 110.7383W 597m, 17 March 2011, J. Schaller	Wide rocky/sandy bank along Gila River, some mesquite and cottonwood, surrounding hills with Sonoran desertscrub	10 (139)
**Site 4**	USA: AZ: Pima Co., Sabino Canyon, Bluff Trail, 33.8991N, 111.4843W, 1098m, 30 April 2011, J. Schaller	Sandy/rocky perennial creek with extensive vegetation, mostly ash and willow, surrounding hills with Sonoran desertscrub	7 (34)
**Site 5**	USA: Arizona: Cochise Co. San Pedro River near Hwy. 82, 31.71358N, 110.18986W, 1176 m, 1 June 2011, J. Schaller	Wide sandy/silty intermittent riverbed of San Pedro River, some large cottonwoods, few rocks	3 (24)
**Site 6**	USA: AZ: Santa Cruz Co., Pajarito Mts, Sycamore Canyon, 31.43195N, 111.18895W, 1191m, 13 March 2010, J. Schaller	Rocky intermittent creek with extensive vegetation, walnut, ash, cottonwood, and willow, surrounding hills with oak woodland	12 (244)
**Site 7**	USA: AZ: Santa Cruz Co., Atascosa Mts., Pena Blanca Lake, 31.3986N, 111.0896W, 1170m, 7 November 2010, J. Schaller	Rocky intermittent creek meeting with Lake Pena Blanca, grassy with *Acacia*, surrounding hills grassy with oak woodland	9 (504)

Collection data and site descriptions for the seven collecting localities sampled in this study. The total number of aggregations and the total number of *Brachinus* individuals (in parentheses) collected at each site are indicated in the last column.

Species presence and abundance were recorded for each site and each aggregation. Specimens of each species were selected for molecular phylogenetic analysis. Using absolute abundance measures for each species within each aggregation, we asked if any two species showed a preference to settle together. Because there were significant differences in the overall abundances of the species across all sites, we tested the three most abundant species (*B*. *elongatulus*, n=720; *B*. *mexicanus*, n=192; *B*. *hirsutus*, n=177) with a parametric Pearson’s correlation and the five least abundant species (*B*. *importitis*, n=29; *B*. *favicollis*, n=16; *B*. *costipennis*, n=3; *B*. *gebhardis*, n=3; *B*. *lateralis*, n=4) with a non parametric Spearman’s Rho correlation. We also used randomization tests to evaluate the likelihood that individuals settled at random in aggregations with respect to the identity and relative abundance of species at a site. Such tests were conducted for sites 2, 3, 6 and 7 where ≥ 139 individuals were collected (Table 1). For each aggregation of size n, n individuals were sampled with replacement 10,000 times from the population of individuals collected at a site, and the conditional probability of obtaining the observed species with and without individuals was calculated. We compared the observed and expected number of individuals per species to elucidate the cause (e.g., more species than expected) of nonrandom aggregations. The expected number of individuals per species was calculated by multiplying the proportion of individuals of each species at a site by n. We used results from all randomization tests and a binomial test to evaluate the hypothesis that nonrandom aggregations occurred more often than expected due to type 1 errors (α = 0.05). Specifically, we tested the hypothesis that the proportion of nonrandom aggregations was higher than 0.05, the proportion of randomization tests expected to yield a P value ≤ 0.05 if individuals actually settled at random in aggregations. We used logistic regression for binomial counts to compare the odds of aggregations with more species than expected, less species than expected, and of nonrandom aggregations that occurred for other reasons, followed by a contrast to more specifically compare the odds of aggregations with more and less species than expected.

### Dynamics of aggregation formation

**Fig 4:**
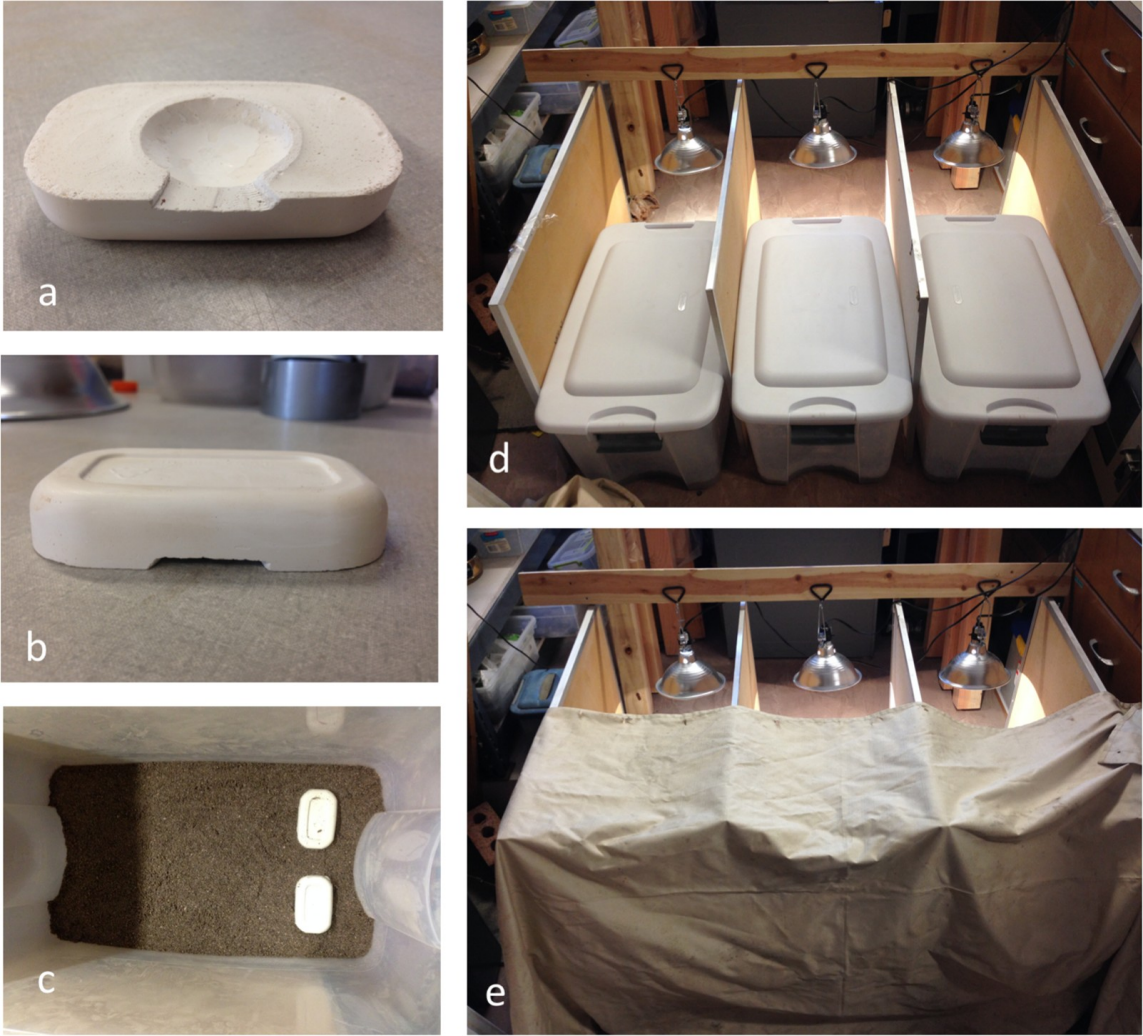
Plaster shelters and arenas for aggregation experiments. (a) Bottom of shelter showing amphitheater and entrance. (b) Front view of shelter showing entrance. (c) Arena with moistened sand substrate. Shelters positioned on the south side of the arena with their entrances facing north. (d) Three arenas set up with separators and daylight simulating bulbs hanging to the north. (e) Opaque curtain covering the south side of arenas.

A video camcorder was placed above an enclosure with a clear cellophane cover to allow for viewing and to maintain humidity. The enclosure was a clear plastic tub (66 x 36 x 33 cm) with 2 cm of moistened substrate (Sakrete™ 15160 multi-purpose sand) on the bottom. To ensure the substrate was consistent for all trials, 2.1 L of sand and 275 mL of water were used for each trial. Plaster of Paris shelters (4 x 6 x 2.5 cm) were constructed with a round cavity on the bottom (3.5 cm in diameter and 1 cm deep) to form an amphitheater for individuals to aggregate (Fig 4A). One entrance (3 cm wide and 5 mm tall) was carved in each shelter leading into the amphitheater (Fig 4B). Shelter bottoms were sanded so that they laid flat on the substrate. Two experiments were video recorded: one with a single unseeded shelter (28 individual *B*. *elongatulus* used), and one with three identical shelters placed side-by-side at one end of the arena with their entrances facing the other end (40 individual *B*. *elongatulus* used). In the second experiment, one shelter was seeded with five tethered conspecifics (left), one was seeded with five tethered congeners (*B*. *mexicanus*, right), and one positioned between them was left empty. Beetles were tethered with a Duncan loop slipknot made of cotton thread and secured around the gap between the prothorax and mesothorax to minimize the effect on mobility.

In both experiments, individual *B*. *elongatulus* were introduced to the shelter at 8 p.m., and the video camcorder was turned on at 3:15 a.m. and left to record until noon. A red light was turned on the entire night (starting at 8:00 p.m.) to illuminate the arenas for recording. A daylight-simulating light was hung above the ground directly north of the arena (opposite of the shelters) and plugged into a timer set for 4:15 a.m. (shortly before actual sunrise) to ensure all shelters were equally illuminated. Videos were analyzed in iMovie® at 8X normal speed. The population of each shelter was assessed over time by tallying every time an individual entered or exited an individual shelter until all the beetles had settled, at which point the aggregations were considered complete.

### Attraction to aggregations of conspecifics and heterospecifics

To test the response of individuals to existing aggregations, we conducted four behavioral experiments. Two experiments were designed to assess whether individuals of a given species preferred to settle with existing aggregations over empty, but otherwise identical, shelters. Two others were designed to assess whether individuals of one species, when given a choice, preferred to settle with groups of their own species (conspecifics) or groups of another species (heterospecifics). The four experiments differed in the species identity of the test individuals (those allowed to choose between shelters), the number of shelters with, and the species identity of, tethered individuals (Table 2).

**Table 2:** Details of four choice experiments.

	n	Test species	Shelter Choice 1	Shelter Choice 2
**Experiment 1**	32	*B*. *elongatulus*	5 tethered *B*. *elongatulus*	empty shelter
**Experiment 2**	25	*B*. *elongatulus*	5 tethered *B*. *hirsutus*	empty shelter
**Experiment 3**	34	*B*. *elongatulus*	5 tethered *B*. *elongatulus*	5 tethered *B*. *hirsutus*
**Experiment 4**	24	*B*. *hirsutus*	5 tethered *B*. *elongatulus*	5 tethered *B*. *hirsutus*

Details of four choice experiments, including the number of trials conducted, the test species and the two shelter choices for each. n= number of trials

The same enclosure and plaster shelter design were used in all experiments (Fig 4). Two shelters were placed side-by-side 4 cm apart on the south side of each enclosure with their entrances facing north (Fig 4C). Each enclosure was covered by an opaque white plastic lid (to control for humidity and light) and placed in between two plywood boards with a daylight-simulating light (programmed to turn on at 4:15 a.m., just before sunrise) suspended above the middle of the north-facing side (Fig 4D), such that an even amount of light illuminated each enclosure from the north. The south sides of the enclosures were covered with an opaque curtain (Fig 4E). Again, this was to ensure that the shelters were equally illuminated, preventing any light biases.

At 9 p.m. (when the beetles are naturally actively foraging/wandering), 25 individuals were placed in the center of each enclosure. The enclosures were then left undisturbed until all 25 individuals had aggregated (usually by around 4:30 a.m. to 5:30 a.m.). The next morning, shelters were lifted to reveal how many individuals chose the different shelters. Numbers were recorded for several trials of the four different choice experiments (see Table 2). Every trial used fresh field collected specimens, newly made plaster shelters, and fresh sand. For all trials, a random number generator was used to determine the positions (left or right) of the two shelters. For each experiment, the mean proportion of beetles settling in a particular shelter type (e.g., with 5 tethered *B*. *elongatulus* individuals in experiment 1) was calculated across trials. We used the bootstrap method with 10,000 repetitions to calculate the 95% confidence interval associated with this mean proportion. A mean proportion greater than 0.5 (the proportion expected without a settling preference) and a 95% confidence interval not overlapping 0.5 was taken as evidence that beetles preferred to settle in a particular shelter type.

## Results

### Molecular phylogenetic analysis

**Fig 5:**
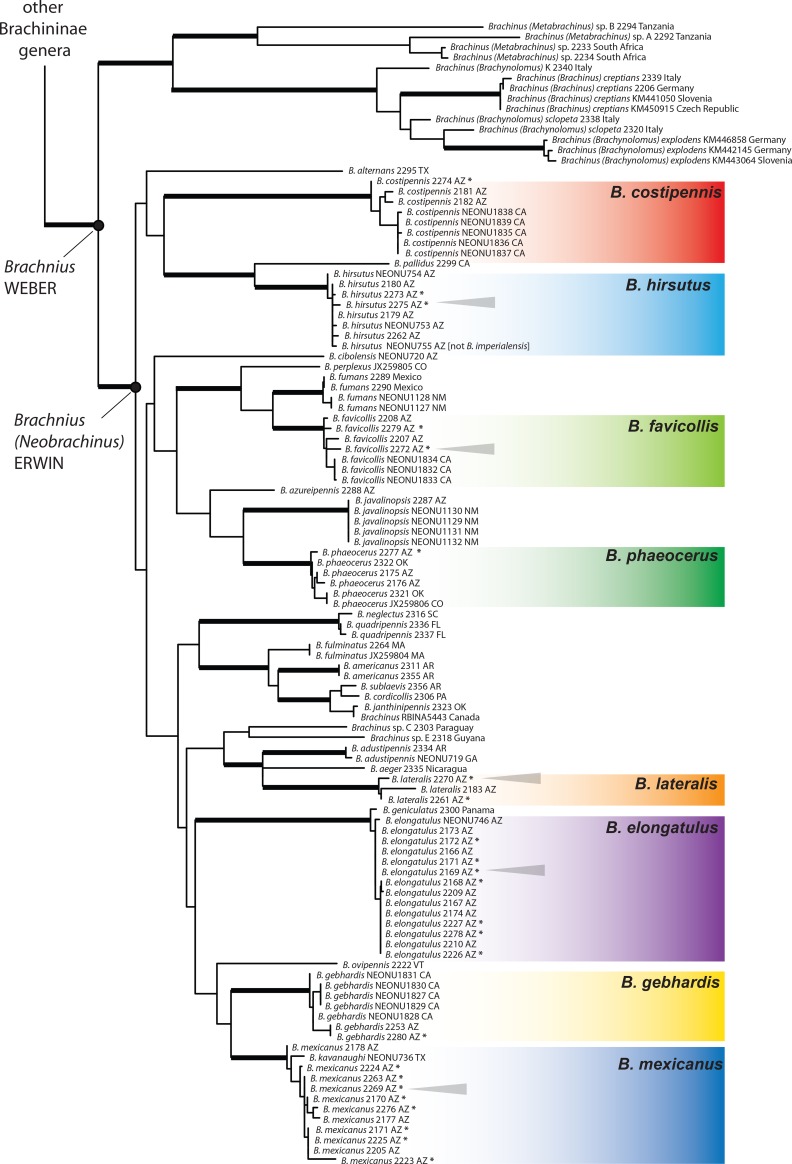
Maximum likelihood tree of concatenated matrix. Outgroups not shown. Branches with bootstrap values >90 are thickened. The eight species found in multispecies aggregations in Arizona are highlighted in color. Asterisks denote specimens collected from multispecies aggregations. Grey triangles point to five species found in a single aggregation at Site 3.

Our molecular phylogenetic analyses revealed strong support for the monophyly of *Neobrachinus* Erwin, a subgenus of *Brachinus*, which includes all 62 species endemic to the Western Hemisphere (Fig 5). Eight of the 16 species that occur in central and southern Arizona participate in multispecies aggregations. These eight species are widely distributed throughout the tree (the closest relatives of each of the eight species live in other regions of North and South America, where multispecies aggregations have not yet been documented). The molecular-based phylogeny confirmed our species identifications, initially based on morphology, showing that eight species were collected in the multispecies aggregations including *Brachinus elongatulus*, *B*. *mexicanus* DEJEAN, *B*. *hirsutus*, *B*. *favicollis* ERWIN, *B*. *lateralis* DEJEAN, *B*. *costipennis* MOTSCHULSKY, *B*. *gebhardis* ERWIN, and *B*. *phaeocerus* CHAUDOIR. The phylogeny revealed no evidence of species hybridization (Fig 5).

### Field collections

**Fig 6:**
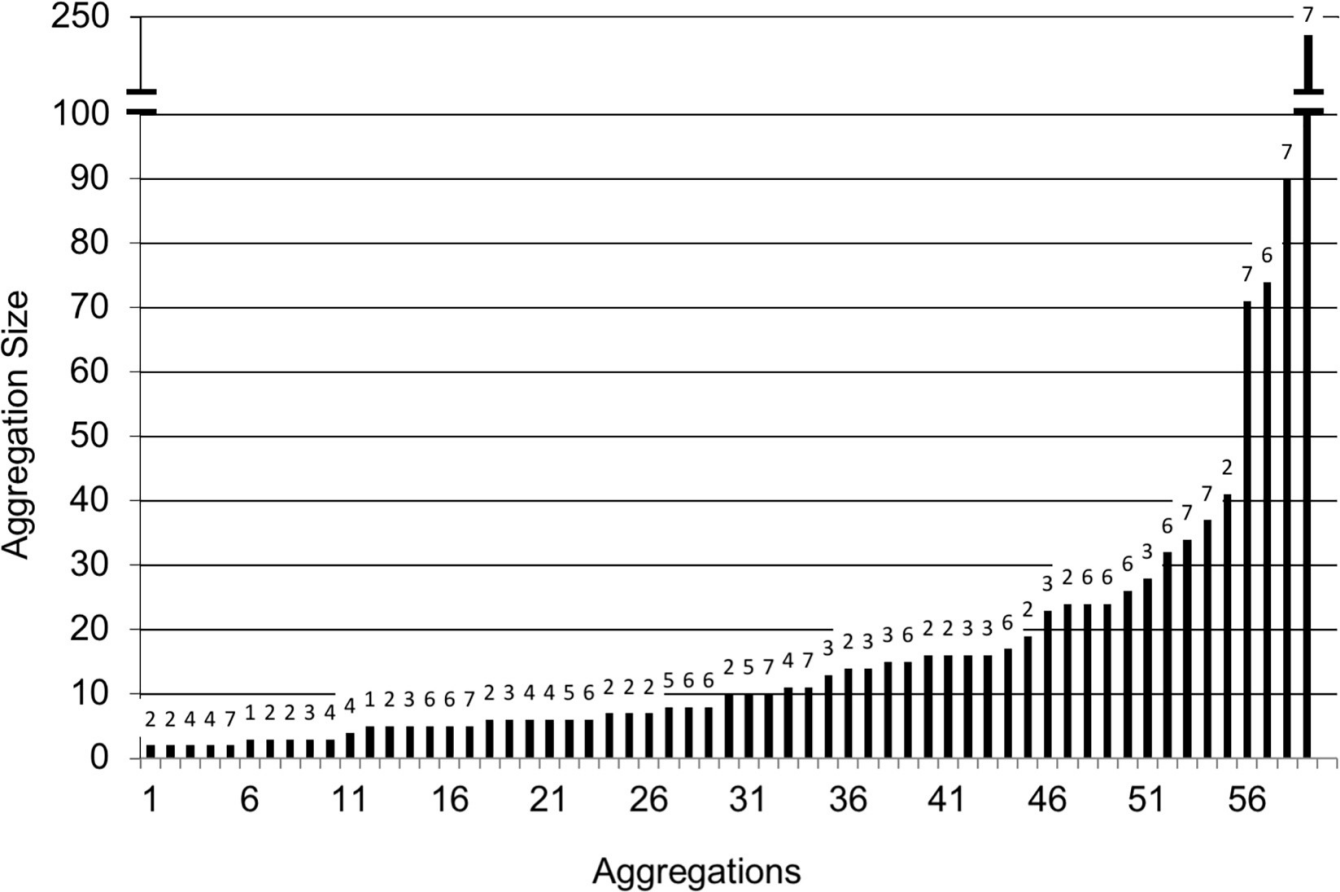
Aggregation size distribution. Size distribution of all 59 aggregations collected ordered from smallest to largest; average 19.2; median 10. Numbers above bars represent the collecting site (1-7) of each aggregation as shown in Table 1.

Fifty-nine aggregations were collected from seven sites totaling 1144 individuals (Table 1, Fig 6). The aggregations ranged in size from two to 248 individuals (avg. = 19.2). Lone individuals were seldom found, and the few that were seen (17 occurrences) were disregarded. Other carabids (*Chlaenius*, *Agonum*, *Platynus*, *Panageus*, *and Galerita)* and/or other arthropods (isopods, spiders, and non-carabid beetles) occurred in roughly one third of the aggregations.

**Fig 7:**
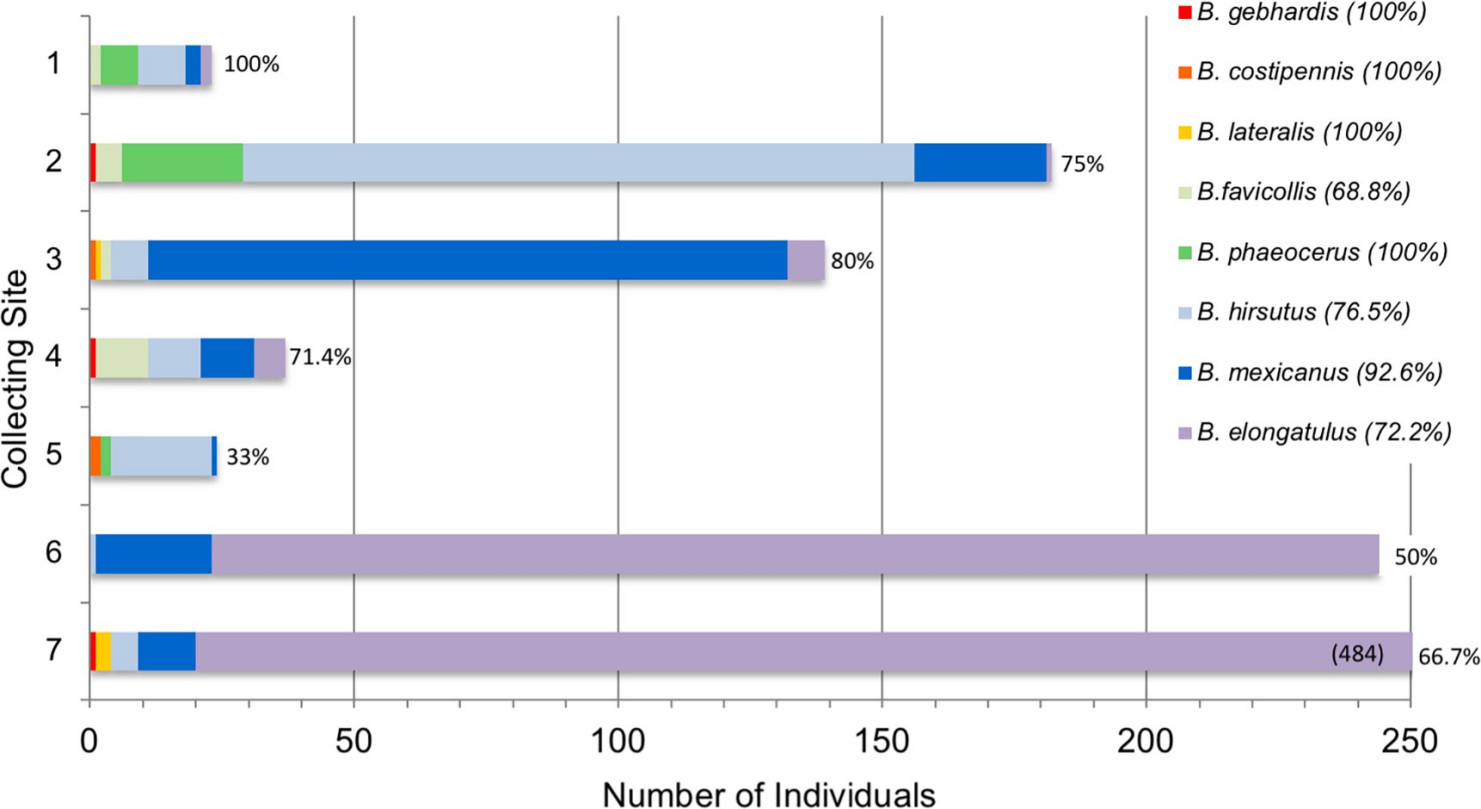
Species content by site. Species content at each collecting site. The percent of aggregations that contained multiple species is indicated to the right of each bar. Percentages of individuals of each species found in multispecies aggregations are next to the species names in the legend. We collected 484 individuals at Site 7, the bar was truncated in this figure to aid visual comparison among sites.

Site-wide composition and relative abundance of *Brachinus* species differed among sites (Fig 7). *B*. *hirsutus* was most common at Sites 2 and 5 (69.8% and 79% respectively), *B*. *mexicanus* most common at Site 3 (87.1%), and *B*. *elongatulus* most common at Sites 6 and 7 (90.6% and 96.0% respectively). However, not all sites had a dominant species. There were roughly even numbers of two co-dominant species at Site 1 and of four co-dominant species at Site 4.

**Fig 8:**
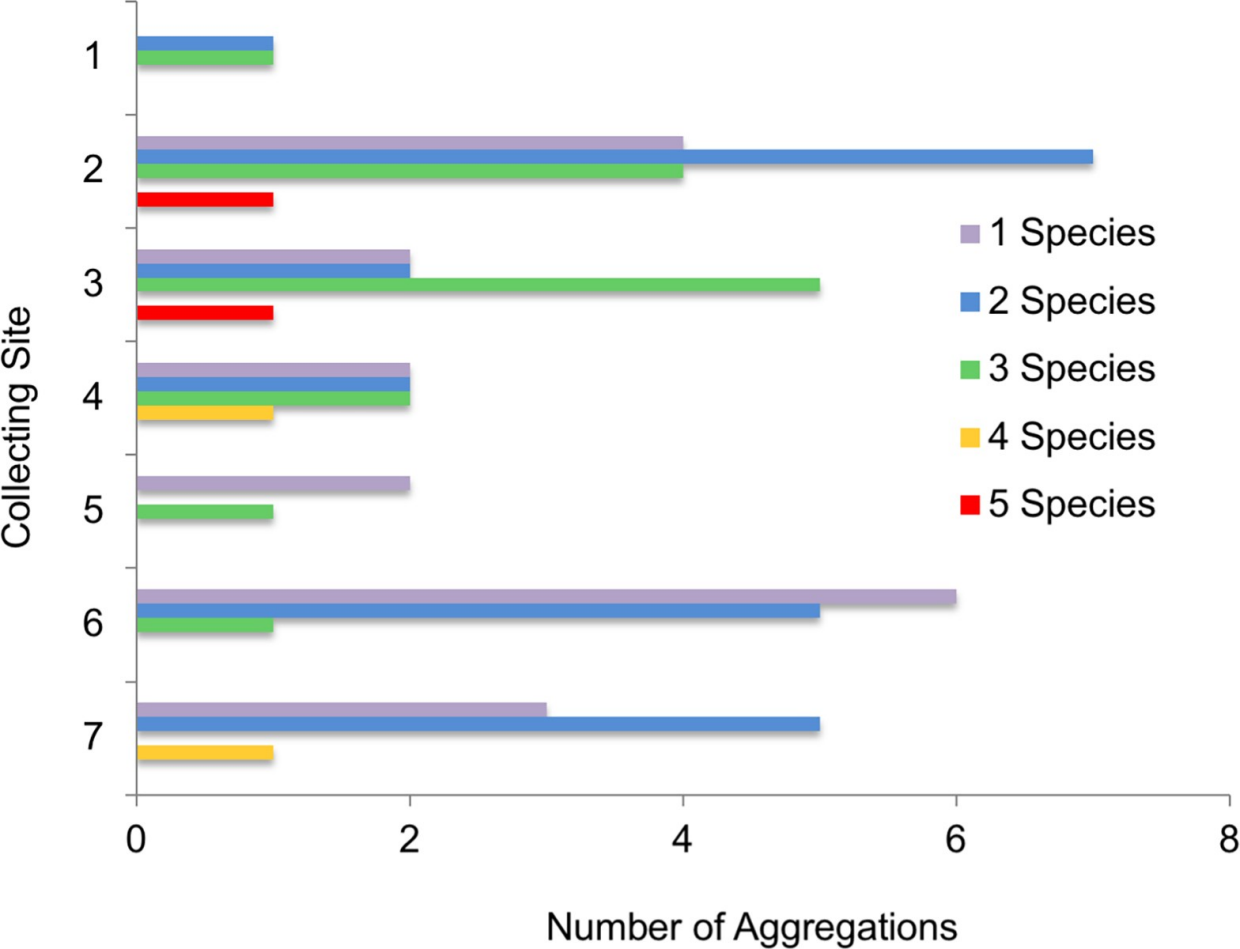
Aggregation type by site. Number of single and multispecies aggregations found at each collecting site.

**Fig 9:**
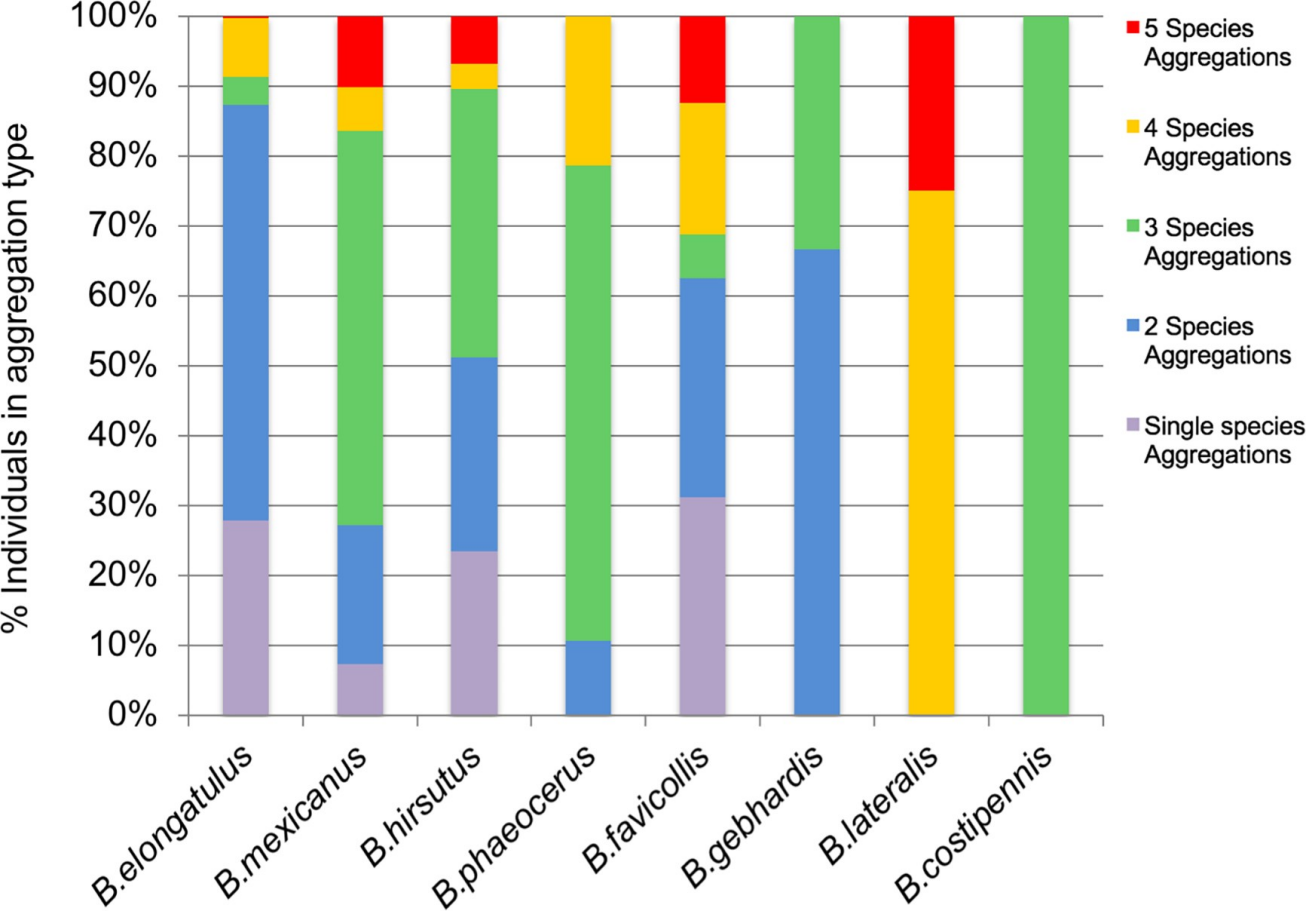
Species participation by aggregation type. Percentage occurrence of each species by aggregation type.

Of the 59 aggregations, 43 (71%) contained more than one *Brachinus* species and 17 (28%) contained three or more species (Fig 8). Sites 6 and 7, dominated by *B*. *elongatulus* (Fig 7), had higher proportions of single species aggregations (Fig 8). Even though Site 5 had a dominant species, *B*. *hirsutus*, (Fig 7), no single species aggregations were collected at that site (Fig 8). Throughout the study, all eight species were commonly found in multispecies aggregations, and only four of the eight species were ever found to occur in single species aggregations (Fig 9).

All correlation tests, both the parametric (Table 3) and non parametric (Table 4), were not significant, indicating that a given *Brachinus* species does not show a bias towards co-occurring with any other species in aggregations.

**Table 3:** Results of the Pearson’s correlation tests.

	Species A	Species B	r	n	Lower CI	Upper CI	P
**Comparison 1**	*B*. *mexicanus*	*B*. *elongatulus*	-0.097	720	-0.34	0.16	0.46
**Comparison 2**	*B*. *hirsutus*	*B*. *elongatulus*	-0.19	192	-0.42	0.064	0.14
**Comparison 3**	*B*. *hirsutus*	*B*. *mexicanus*	-0.064	177	-0.31	0.19	0.62

Results of the Pearson’s correlation tests among the three most abundant species. Abbreviations: R- Pearson’s correlation, n= species total abundance across all sites, lower CI and upper CI refer to 95% confidence intervals, P= P-value.

**Table 4:** Results of the Spearman’s rank correlation tests.

	Species A	Species B	Spearman ρ	Prob>|ρ|
**Comparison 1**	*B*. *favicollis*	*B*. *imporcitis*	-0.057	0.66
**Comparison 2**	*B*. *costipennis*	*B*. *imporcitis*	0.19	0.15
**Comparison 3**	*B*. *costipennis*	*B*. *favicollis*	-0.081	0.53
**Comparison 4**	*B*. *gebhardis*	*B*. *imporcitis*	-0.094	0.47
**Comparison 5**	*B*. *gebhardis*	*B*. *favicollis*	-0.10	0.44
**Comparison 6**	*B*. *gebhardis*	*B*. *costipennis*	-0.042	0.75
**Comparison 7**	*B*. *lateralis*	*B*. *imporcitis*	-0.076	0.56
**Comparison 8**	*B*. *lateralis*	*B*. *favicollis*	0.15	0.26
**Comparison 9**	*B*. *lateralis*	*B*. *costipennis*	-0.034	0.79
**Comparison 10**	*B*. *lateralis*	*B*. *gebhardis*	-0.042	0.75

Results of the Spearman’s rank correlation tests for the five least abundant species.

Nevertheless, *Brachinus* individuals often did not aggregate randomly (S2 Table – S5 Table). Across four sites, 34% (16/47) of aggregations were nonrandom, which is significantly higher than expected by chance (binomial test, P < 0.0001). Seventeen percent (8/47) of the aggregations were nonrandom and contained more species than expected, 8.4% (5/47) were nonrandom and contained less species than expected, and 6.4% (3/47) were nonrandom for other reasons. However, the odds of nonrandom aggregations with more species than expected did not differ significantly from the odds of aggregations with less species than expected (χ^2^ = 0.61, P = 0.43).

### Dynamics of aggregation formation

**Fig 10:**
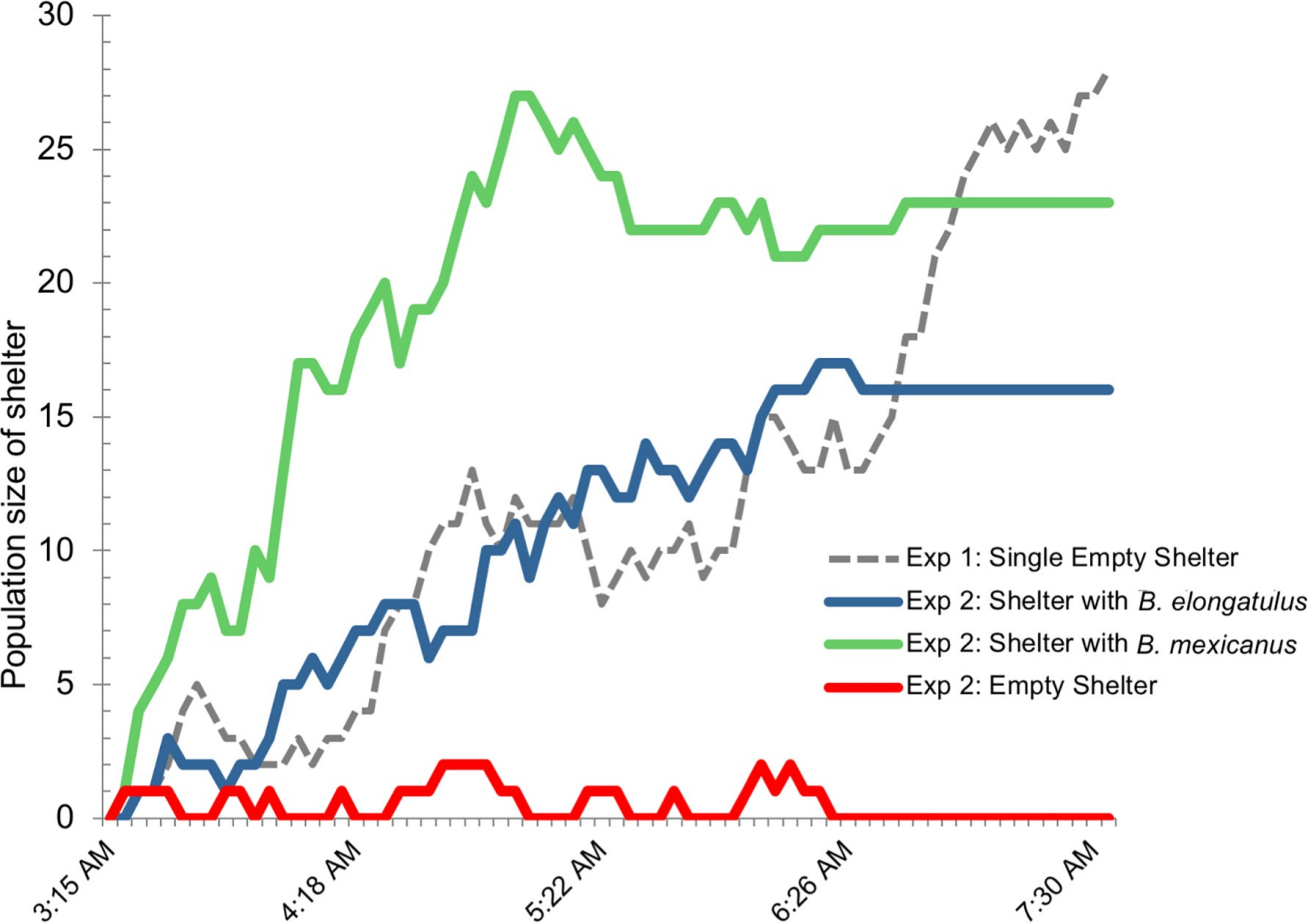
Dynamics of aggregation formation. Timing of aggregation growth of *Brachinus elongatulus* during two experiments. In both experiments the daylight-simulating bulb came on at 4:15 AM. Experiment 1: beetles with one shelter to aggregate under (grey dotted line). Experiment 2: beetles with a choice of a shelter with five tethered conspecifics (blue line); a shelter with five tethered *B*. *mexicanus* (green line); and an empty shelter (red line).

Video recordings of aggregations formed in a laboratory arena revealed the numbers of individuals in the shelter(s) fluctuated continuously as individual *B*. *elongatulus* entered and exited (Fig 8). During this process, individuals never traveled together or followed each other outside of the shelters, but appeared to act individually, exploring the shelter and leaving several times before finally remaining in the shelter. It took about 5 hours (roughly between 3:00 and 8:00 a.m.) for all individuals to settle in the shelter in the single-shelter trial, and it took 4 hours (between 3 and 7 a.m.) for all individuals to settle in a shelter in the three-shelter trial. In the three-shelter trial, individuals were divided between the shelters seeded with *B*. *elongatulus* versus *B*. *mexicanus*, both shelters taking about the same time to become fully settled (Fig 10). No individuals settled in the empty shelter.

### Attraction to existing aggregations

*B*. *elongatulus* significantly preferred to settle in shelters already seeded with *Brachinus* individuals over settling in empty shelters. The average proportion of *B*. *elongatulus* settling in a shelter with conspecifics vs. an empty shelter was 0.59 (95% confidence interval = 0.51 – 0.67), whereas the average proportion of *B*. *elongatulus* settling in a shelter with heterospecifics vs. an empty shelter was 0.68 (95% confidence interval = 0.56 – 0.80). When given a choice of a shelter seeded with conspecifics versus heterospecifics, *B*. *elongatulus* showed a tendency to shelter with heterospecifics but the difference was not statistically significant (mean proportion sheltering with heterospecifics = 0.55, 95% confidence interval = 0.49 – 0.61). However, *B*. *hirsutus* did show a significant preference to settle in shelters seeded with *B*. *elongatulus* over those seeded with conspecifics (mean proportion sheltering with heterospecifics = 0.65, 95% confidence interval = 0.58 – 0.71).

## Conclusions

Our molecular phylogeny shows that individuals of eight species of *Brachinus* that commonly form multispecies aggregations in riparian habitats in central and southeastern Arizona show no signs of hybridization (Fig 3). It stands to reason that effective reproductive isolating mechanisms among the species involved may be a prerequisite for multispecies aggregations among congeners to exist and persist through time as this would allow for a genus-wide aggregating cue to exist with little effect on species boundaries. That the Arizona *Brachinus* are not each other’s closest relatives (Fig 3) suggests that these species may have evolved in different places via allopatric speciation and only later became sympatric. However, our observations of larval host preferences and of adult behavior in the field also point to the possibility that host shifts and shifts in temporal distributions of reproductive activity may have also played a role in speciation. In North America, *Brachinus* spp. larvae are parasitoids on the pupae of Dytiscidae, Hydrophilidae, and Gyrinidae water beetles [18, 29–33]. First instar *Brachinus* larvae are highly mobile and actively search for aquatic beetle pupae. When found, the *Brachinus* larva consumes the entire water beetle pupa while progressing through its own larval development to pupation, a process that takes approximately one month [29, 30]. Because host species have different temporal and spatial distributions, this is likely reflected in the timing of the parasites’ life histories as well.

While collecting individuals at night along the San Pedro River, were at least 10 species of *Brachinus* co-occur, we observed two years in a row that *B*. *hirsutus* were the only individuals seen mating during the month of April. And during the month of June, there were large numbers of teneral (freshly eclosed) *B*. *hirsutus* adults, suggesting a recently completed life cycle. Because the life cycle of the closely related *B*. *pallidus* has been shown to take roughly a month from egg to pupa [18], it makes sense that *B*. *hirsutus*, at least at this particular location, has a breeding season in April that yields June adults. In October at the same location, only *B*. *mexicanus* were observed mating, even though *B*. *hirsutus* adults were also present in large numbers. It is likely that the timing of *B*. *hirsutus* and *B*. *mexicanus* life cycles coincide with specific host species that enter their pupal stages at different times [18, 32–35]. A similar host synchrony was observed in European *Brachinus* species parasitizing pupae of *Amara;* both *B*. *crepitans* and *B*. *explodens* were shown to commence reproduction in the presence of specific *Amara* pupae but not when exposed to other pupae [34]. If host shifts drove speciation in *Brachinus*, different timing of the host life cycles may help maintain reproductive isolation and species boundaries among sympatric *Brachinus* species today.

The vast majority of the *Brachinus* individuals collected in this study, regardless of their species identity, were found as members of multispecies aggregations (Figs 4, 6 and 7). Species-pair correlation tests showed no two *Brachinus* species were more commonly found together in an aggregation than would be expected based on their relative abundance in the population (Tables 3 and 4). Randomization tests showed that species composition of the aggregations was often nonrandom (S2–S5 Tables). Although 17 and 8.4% of the aggregations were nonrandom and respectively contained more or less species than expected, these percentages were not significantly different, indicating no tendency of *Brachinus* individuals to form aggregations with a higher number of species than expected based on the species relative abundance. Nevertheless, results of laboratory experiments indicate that beetles show a strong preference to settle in shelters that are occupied by congeners. Furthermore, the two *Brachinus* species tested did not prefer to shelter with conspecifics over heterospecifics, and one species, *B*. *hirsutus*, *clearly* preferred to shelter with *B*. *elongatulus*. Both the lack of discrimination by *B*. *elongatulus* and the heterospecific preference by *B*. *hirsutus* should contribute to formation of multispecies aggregations.

### Mechanisms

Relatively little is known about how multispecies aggregations form. Do individuals of one species actively join groups containing other species, or are multispecies aggregations simply a consequence of individuals of each species settling together in response to the same local environmental cues? To date, little information as to what cues influence formation of *Brachinus* aggregation has been available. In our video recordings of aggregation formation, individual beetles appear to act alone, that is, they moved alone rather than in tandem as they sought shelters, and they were already exploring shelters when the recordings began around 3:15 a.m. Aggregations began to grow well before the simulated daylight bulb came on at 4:15 a.m., and the individuals repeatedly entered and exited all shelters in the arena before settling (Fig 10). These observations suggest that the cue to start seeking shelter is not determined by light, but by a circadian clock. This idea is supported by activity cycles observed in cave beetles (*Rhadine* sp.), in which individuals became active only at night despite living in a zero-light habitat (J. Reddell, pers. comm.).

Chemical cues have been shown to stimulate aggregation in other gregarious insects, including specific non-volatile chemicals on the cuticles of some grasshopper species [36] and volatile carboxylic acids produced by gut microbiota found in the feces of the German cockroach, *Blattella germanica* [37]. Whether the cue for *Brachinus* is chemical (volatile or non-volatile) or tactile, it is likely to be effective across all members of the genus, given that the species in Arizona that regularly participate in this behavior are spread throughout the phylogeny of the genus (Fig 5).

In studying intraspecific attraction between individuals of *Brachinus* species in France Wauteir showed that individuals of one species vary in the degree of their attraction to one another, that the variation does not correlate with sex or age, and that the attraction universally wanes for both sexes during reproductive season [11]. Attraction was completely diminished when the antennae of some individuals were experimentally removed, suggesting that the aggregation stimulus is chemical in nature [11].

A study of multispecies aggregations of *Brachinus sclopeta* FABRICIUS and *Anchomenus dorsalis* PONTAPPIDEN (Platynini) in Europe may provide even more insights into the nature of the cue [9]. *Anchomenus dorsalis* individuals rub themselves on individuals of *B*. *sclopeta* before settling with them. Further, *A*. *dorsalis* engaged in rubbing behavior with *B*. *sclopeta* less frequently the longer they were exposed to them. We observed a similar form of desensitization in our study. In early test trails of our experiments, we found that individual *B*. *elongatulus* had a greater tendency to settle in empty shelters over those seeded with conspecifics the longer the test and tethered individuals had been exposed to one another prior to the experiment. The trend diminished when freshly collected *B*. *elongatulus* were subsequently used to seed the shelters. This pattern suggests that individuals may be desensitized to one another, and less attracted to them, the longer they have been exposed to one another. Of course, this type of desensitization could occur with any stimulus—tactile, visual, vibratory, as well as chemical. However, *A*. *dorsalis* also rub themselves on filter paper that had been swabbed with *B*. *sclopeta* [9], suggesting that a chemical cue is involved in that system.

### Functional significance

Our research suggests that there may be functional consequences of individuals settling in groups containing multiple species. This may simply be that multiple species can form larger aggregations together than they could if just one species participated, resulting in a larger benefit of aggregation, such as enhanced aposematic signaling [38], greater dilution effect or the group chemical defense being more potent simply because there are more individuals in the group.

We speculate that, in the case of *Brachinus*, the evolutionary significance of multispecies aggregations is more related to predator avoidance than it is to group chemical defense, as *Brachinus* individuals do not spray “en masse” when their shelter is disturbed, but rather each individual sprays only after it has been physically attacked. This reluctance to spray was observed in chemically defended whirlgig beetles which form large dense “rafts” of hundreds to thousands of individuals consisting of multiple species on the water surface during the day [8]. However, being a member of a large aggregation would protect individuals that have recently sprayed. After an individual *Brachinus* sprays to exhaustion, they must wait 24-36 hours to recharge their chemical stores to effectively fire again (WM, pers. obs.). It is interesting that all four of the known examples of multispecies aggregations in arthropods occur in taxa that are chemically defended (harvestmen, whirligig beetles, lycids, and carabid beetles). It is possible that, in the case of these lineages, chemical defense is costly and large aggregations decrease the likelihood that any one individual would need to use its arsenal at any given time. And those individuals that do fire, are protected by their proximity to other individuals that are still able to fire within in the aggregation. Harvestmen (Arachnida: Opiliones) form aggregations of three species in Brazilian swamps [5]. Although all three species are chemically defended, in response to disturbance only one of the three species regularly released defensive chemicals, which immediately resulted in individuals leaving the group and fleeing in different directions. It was hypothesized that all three species benefit from the aggregation: two species get protection from a third species’ chemical defense, and all three species take advantage of a dilution effect [5].

*Brachinus* aggregations may serve to reduce per capita predation risk. Both sexes of all *Brachinus* species produce defensive benzoquinones but the fine-scale composition of defensive spray and/or the beetles’ readiness or reluctance to spray remains untested, and may vary among species. One study found slight differences in the shape and size of the reactions chambers between males and females of *B*. *elongatulus* [39] which might result in a sexual difference of their spray. However, if both sexes of all *Brachinus* species do indeed have equal powers to punish and educate predators, then all individuals of both sexes in all species can contribute equally to the defense of the group and education of the group’s predators. If this is true, then there should be no selection favoring the rejection of any *Brachinus* species of either sex from joining an aggregation. Likewise, there should be no selection for preferences of either sex or any species. The dilution effect should create individual selection for all *Brachinus* to always welcome all joiners, and strong individual selection on all *Brachinus* to be joiners. This seems to stand in contrast to one of the results of our behavioral assays, in which individuals of *B*. *hirsutus* actually preferred to shelter with *B*. *elongatulus* (Fig 10B). However, all beetles for that study were collected from a site along the San Pedro River where *B*. *elongatulus* is by far the most abundant species. It could be that less common species at any one site are conditioned to be attracted to the common species to yield larger, safer aggregations.

Three results of this research, when taken together, give it general significance for the study of social behavior. We present the first phylogenetic analysis of congeners that participate in multispecies aggregations, and find that the species found in aggregations are not each other’s closest relatives but rather are dispersed throughout the phylogeny of the genus (Fig 3). Further we find no tendency for species to aggregate with close relatives over more distant relatives. Second, we show that the beetle aggregations, including the multispecies component, involve an active preference on part of beetles to aggregate. And finally, we show that at least one *Brachinus* species has a preference to shelter with a different species, suggesting multispecies aggregations are actively formed. This research lays the groundwork for future explorations into the nature of multispecies aggregation behavior within this group and among terrestrial arthropods in general. Assessing which chemical cues play a role in *Brachinus* aggregation, and assessing the function and evolutionary benefits of heterospecific groups, will be important next steps in our understanding of the evolution and fascinating behavior of these bombardier beetles.

## Supporting information

S1 Table Taxon sampling. Taxonomic and geographic information for the specimens used in this study and GenBank accession numbers for each sequence.
(DOCX)

S2 Table Observed number of individuals per *Brachinus* species in the 16 aggregations collected at Site 2. Parentheses show the expected number of individuals per species in each aggregation if all individuals settle at random with respect to the identity and relative abundance of species collected at Site 2. P value is the probability of finding the observed number of species in each aggregation based on a randomization test.
(DOCX)

S3 Table Observed number of individuals per *Brachinus* species in the10 aggregations collected at Site 3. Parentheses show the expected number of individuals per species in each aggregation if all individuals settle at random with respect to the identity and relative abundance of species collected at Site 3. P value is the probability of finding the observed number of species in each aggregation based on a randomization test.
(DOCX)

S4 Table Observed number of individuals per *Brachinus* species in the12 aggregations collected at Site 6. Parentheses show the expected number of individuals per species in each aggregation if all individuals settle at random with respect to the identity and relative abundance of species collected at Site 6. P value is the probability of finding the observed number of species in each aggregation based on a randomization test.
(DOCX)

S5 Table Observed number of individuals per *Brachinus* species in the nine aggregations collected at Site 7. Parentheses show the expected number of individuals per species in each aggregation if all individuals settle at random with respect to the identity and relative abundance of species collected at Site 7. P value is the probability of finding the observed number of species in each aggregation based on a randomization test.
(DOCX)
