# Supplementary material for: Molecular phylogeny, ecology and multispecies aggregation behaviour of bombardier beetles in Arizona
Source: PLoS One. 2018 Oct 31;13(10):e0205192. doi: 10.1371/journal.pone.0205192 (PMC6209175; doi:10.1371/journal.pone.0205192)
Supplement: S1 Table — Taxonomic and geographic information for the specimens used in this study and GenBank accession numbers for each sequence. (DOCX) [file pone.0205192.s001.docx]

| **Voucher Species** | **Voucher Number** | **Location and Collecting Data** | **CAD GenBank Accession Number** | **CO1 GenBank Accession Number** |
| --- | --- | --- | --- | --- |
| *Pheropsophus* (*Aptinomorphus*) | 2195 | MADAGASCAR: Toamasina Prov., Masoala Nat. Park, 50-300m, 14-17 Dec 2008, Coll. W. Moore |  | KU937720 |
| *Pheropsophus* (*Pheropsophus*) | 2230 | SOUTH AFRICA: Zwa-Zulu Natal, St.Lucia, 17m, 16 Jan 2004, W. Moore | KU937658 | KU937730 |
| *Styphlomerus* | 2231 | SOUTH AFRICA: Limpopo, Hans Merensky NR, 401 m, 16 Dec 2003 W. Moore | KU937659 | KU937731 |
| *B.* (*Brachinus*) *crepitans* | 2206 | GERMANY: Thuringia, Kullstedt, 7 May 2010 | KU937647 | KU937721 |
| *B.* (*Brachinus*) *crepitans* | 2339 | ITALY: Lazio: Acropoli di Tarquinia (vr), 10 Mar 2013, coll. M. Bologna | KU937697 |  |
| *B.* (*Brachynolomus*) *sclopeta* | 2320 | ITALY: Sicily, Palermo Prov., Piano Battaglia, Parc Regione delle Madonie, 1560m, 11 May 2011, D. Brzoska | KU937688 | KU937764 |
| *B.* (*Brachynolomus*) *sclopeta* | 2338 | ITALY: Lazio: Acropoli di Tarquinia (vr), 10 Mar 2013, coll. M. Bologna | KU937696 |  |
| *B.* (*Brachynolomus*) *sp. K* | 2340 | ITALY: Lazio: Acropoli di Tarquinia (vr), 10 Mar 2013, coll. M. Bologna | KU937698 |  |
| *B.* (*Metabrachinus*) *sp. A* | 2292 | TANZANIA: Morogoro, Mvomelo District, Milama, 13 Nov. 2011, coll. K. Angelus | KU937679 | KU937755 |
| *B.* (*Metabrachinus*) *sp. B* | 2294 | TANZANIA: Morogoro, Mvomelo District, Milama, 13 Nov. 2011, coll. K. Angelus, Lot #KA13-XI-2011 | KU937681 | KU937756 |
| *B.* (*Metabrachinus*) *sp. D* | 2233 | SOUTH AFRICA: Mpumalanga, Berlin Forest, 920m, 14 Dec 2003, coll. W. Moore. | KU937660 | KU937732 |
| *B.* (*Metabrachinus*) *sp. D* | 2234 | SOUTH AFRICA: Limpopo, Waterberg Mts.,1490m, 5 Dec 2003, coll. W. Moore | KU937661 | KU937733 |
| *B.* (*Neobrachinus*) *adustipennis* | 2334 | USA: AR: Montgomery Co., Lake Ouachita, 2 mi N Joplin off US 270, 11 Aug 2010, coll. Davidson, Acciavati, & Baldwin | KU937692 | KU937768 |
| *B.* (*Neobrachinus*) *aeger* | 2335 | NICARAGUA: Granada Prov., Domitila Reserva Silvestre Privada ESE Nandaime, 120m, 7 Jun 2005, coll. C.B. Barr & C.S. Chaboo, | KU937693 | KU937769 |
| *B.* (*Neobrachinus*) *alternans* | 2295 | USA: TX: Potter Co., Plum Creek, 1 Oct 2008, coll. Vaughan, Jones | KU937682 | KU937757 |
| *B.* (*Neobrachinus*) *americanus* | 2311 | USA: AR: Montgomery Co., Little Missouri Falls, 8 Apr 2011, coll. B. Baldwin | KU937686 | KU937762 |
| *B.* (*Neobrachinus*) *americanus* | 2355 | USA: AR: Montgomery Co., Little Missouri Falls, 8 Apr 2011, coll. B. Baldwin | KU937699 |  |
| *B.* (*Neobrachinus*) *azureipennis* | 2288 | USA: Arizona: Cochise Co., San Pedro River near Hwy. 82, 1180 m, 4 Oct 2012, coll. J. Schaller | KU937676 | KU937752 |
| *B.* (*Neobrachinus*) *cordicollis* | 2306 | USA: PA: Lycoming Co. Loyalsock Creek, 4.8km E of Barbours, 230m, 7 May 2003, coll. Matthew J. Kowalski | KU937685 | KU937761 |
| *B.* (*Neobrachinus*) *costipennis* | 2181 | USA: Arizona, Cochise Co., San Pedro River, 1200m, 3 Jun 2004, coll. W.Moore | KU937643 | KU937717 |
| *B.* (*Neobrachinus*) *costipennis* | 2182 | USA: Arizona, Cochise Co., San Pedro River, 1200m, 3 Jun 2004, coll. W.Moore | KU937644 | KU937718 |
| *B.* (*Neobrachinus*) *costipennis* | 2274 | USA: Arizona, Pinal Co. 3 mi NE Winkleman, Gila River, 600m, 17 Mar 2011, coll. J. Schaller | KU937668 | KU937744 |
| *B.* (*Neobrachinus*) *elongatulus* | 2166 | USA: Arizona, Santa Cruz Co., Pajarito Mts., Sycamore Canyon, 1190m, 13 Mar 2010, coll. W.Moore, J. Schaller, R.L. Smith | KU937628 | KU937704 |
| *B.* (*Neobrachinus*) *elongatulus* | 2167 | USA: Arizona, Santa Cruz Co., Pajarito Mts., Sycamore Canyon, 1190m, 13 Mar 2010, coll. W.Moore, J. Schaller, R.L. Smith | KU937629 | KU937705 |
| *B.* (*Neobrachinus*) *elongatulus* | 2168 | USA: Arizona, Santa Cruz Co., Pajarito Mts., Sycamore Canyon, 1190m, 13 Mar 2010, coll. W.Moore, J. Schaller, R.L. Smith | KU937630 |  |
| *B.* (*Neobrachinus*) *elongatulus* | 2169 | USA: Arizona, Santa Cruz Co., Pajarito Mts., Sycamore Canyon, 1190m, 13 Mar 2010, coll. W.Moore, J. Schaller, R.L. Smith | KU937631 | KU937703 |
| *B.* (*Neobrachinus*) *elongatulus* | 2172 | USA: Arizona, Santa Cruz Co., Pajarito Mts., Sycamore Canyon, 1190m, 13 Mar 2010, coll. W.Moore, J. Schaller, R.L. Smith | KU937634 | KU937708 |
| *B.* (*Neobrachinus*) *elongatulus* | 2173 | USA: Arizona, Santa Cruz Co., Pajarito Mts., Sycamore Canyon, 1190m, 13 Mar 2010, coll. W.Moore, J. Schaller, R.L. Smith | KU937635 | KU937709 |
| *B.* (*Neobrachinus*) *elongatulus* | 2174 | USA: Arizona, Santa Cruz Co., Pajarito Mts., Sycamore Canyon, 1190m, 13 Mar 2010, coll. W.Moore, J. Schaller, R.L. Smith | KU937636 | KU937710 |
| *B.* (*Neobrachinus*) *elongatulus* | 2209 | USA: Arizona, Cochise Co. Chiricahua Mts., upper Turkey Creek, 2040m, 30-31 May 2010 coll. J. Schaller | KU937650 | KU937722 |
| *B.* (*Neobrachinus*) *elongatulus* | 2210 | USA: Arizona, Cochise Co. Chiricahua Mts., upper Turkey Creek, 2040m, 30-31 May 2010 coll. J. Schaller | KU937651 | KU937723 |
| *B.* (*Neobrachinus*) *elongatulus* | 2226 | USA: Arizona, Santa Cruz Co., Pajarito Mts., Sycamore Canyon, 1190m, 13 Mar 2010, coll. W.Moore, J. Schaller, R.L. Smith | KU937656 | KU937728 |
| *B.* (*Neobrachinus*) *elongatulus* | 2227 | USA: Arizona, Santa Cruz Co., Pajarito Mts., Sycamore Canyon, 1190m, 13 Mar 2010, coll. W.Moore, J. Schaller, R.L. Smith | KU937657 | KU937729 |
| *B.* (*Neobrachinus*) *elongatulus* | 2271 | USA: Arizona, Pinal Co. 3 mi NE Winkleman, Gila River, 600m, 17 Mar 2011, coll. J. Schaller | KU937665 | KU937741 |
| *B.* (*Neobrachinus*) *costipennis* | 2274 | USA: Arizona, Pinal Co. 3 mi NE Winkleman, Gila River, 600m, 17 Mar 2011, coll. J. Schaller | KU937668 | KU937744 |
| *B.* (*Neobrachinus*) *elongatulus* | 2278 | USA: Arizona, Maricopa Co. Lower Sycamore Creek, off Hwy, 720m, 28 Mar 2011, coll. J. Schaller | KU937672 | KU937748 |
| *B.* (*Neobrachinus*) *favicollis* | 2207 | USA: Arizona: Yavapai Co., T18N, R6W sec. 25, Walnut Creek, 8 Jul 2004, coll. R. Delph | KU937648 |  |
| *B.* (*Neobrachinus*) *favicollis* | 2208 | USA: Arizona: Yavapai Co., T18N, R6W sec. 25, Walnut Creek, 8 Jul 2004, coll. R. Delph | KU937649 |  |
| *B.* (*Neobrachinus) favicollis* | 2272 | USA: Arizona, Pinal Co. 3 mi NE Winkleman, Gila River, 600m, 17 Mar 2011, coll. J. Schaller | KU937666 | KU937742 |
| *B.* (*Neobrachinus*) *favicollis* | 2279 | USA: Arizona, Maricopa Co. Lower Sycamore Creek, off Hwy, 720m 28 Mar 2011, coll. J. Schaller | KU937673 | KU937749 |
| *B.* (*Neobrachinus*) *fulminatus* | 2264 | USA: MA: Harvard Forest (NEON Specimen) | KU937662 | KU937738 |
| *B.* (*Neobrachinus*) *fumans* | 2289 | MEXICO: Sonora, La Colorada, E side of Tecoripa, 410m, 22 Sep 2011, coll. T.R. VanDevender, A.L. Reina-G. | KU937677 | KU937753 |
| *B.* (*Neobrachinus*) *fumans* | 2290 | MEXICO: Sonora, Municipio de Carbo. El Oasis on MEX 15, 7.9km W Carbo, 550m, 16 Oct 2011, coll. T.R. VanDevender. | KU937678 | KU937754 |
| *B.* (*Neobrachinus*) *gebhardis* | 2253 | USA: Arizona, Pima Co., Tanque Verde Creek, trail off Reddington Rd., 860m, 27 May 2010, coll. J. Schaller |  | KU937734 |
| *B.* (*Neobrachinus*) *gebhardis* | 2280 | USA: Arizona, Maricopa Co. Lower Sycamore Creek, off Hwy. 87, 720m 28 Mar 2011, coll. J. Schaller | KU937674 | KU937750 |
| *B.* (*Neobrachinus*) *geniculatus* | 2300 | PANAMA: Rio Manoni, 1 Sep 2006 | KU937683 | KU937759 |
| *B.* (*Neobrachinus*) *hirsutus* | 2179 | USA: Arizona, Cochise Co., San Pedro River, Charleston Bridge, 1200m, 23 Feb 2010, coll. W.Moore, D. and J. Maddison | KU937641 | KU937715 |
| *B.* (*Neobrachinus*) *hirsutus* | 2180 | USA: Arizona, Santa Cruz Co., Pajarito Mts., Sycamore Canyon, 1190m, 13 Mar 2010, coll. W.Moore, J. Schaller, R.L. Smith | KU937642 | KU937716 |
| *B.* (*Neobrachinus*) *hirsutus* | 2262 | USA: AZ: Santa Cruz Co. Atascosa Mts. Pena Blanca Lake, 1170m, 7 Nov 2010 J. Schaller |  | KU937736 |
| *B.* (*Neobrachinus*) *hirsutus* | 2273 | USA: Arizona, Pinal Co. 3 mi NE Winkleman, Gila River, 600m, 17 Mar 2011, coll. J. Schaller | KU937667 | KU937743 |
| *B.* (*Neobrachinus*) *hirsutus* | 2275 | USA: Arizona, Maricopa Co. Lower Sycamore Creek, 720m 28 Mar 2011, coll. J. Schaller | KU937669 | KU937745 |
| *B.* (*Neobrachinus*) *janthinipennis* | 2323 | USA: OK: Custer Co. Foss State Park, Sandy Beach, 06 Oct 2012, coll. G.F. Hevel | KU937691 | KU937767 |
| *B.* (*Neobrachinus*) *javalinopsis* | 2287 | USA: Arizona, Cochise Co., San Pedro River near Hwy. 82, 1180 m, 1 Jun 2011, coll. J. Schaller | KU937675 | KU937751 |
| *B.* (*Neobrachinus*) *lateralis* | 2176 | USA: Arizona, Cochise Co., San Pedro River, Charleston Bridge, 1200m, 23 Feb 2010, coll. W.Moore, D. and J. Maddison |  | KU937735 |
| *B.* (*Neobrachinus*) *lateralis* | 2183 | USA: Arizona, Santa Cruz Co., Patagonia Lake State Park, 1150m, 6 Jun 2004, coll. J.C. Oliver and K.L. Prudic | KU937645 | KU937719 |
| *B.* (*Neobrachinus*) *lateralis* | 2261 | USA: AZ: Santa Cruz Co. Atascosa Mts. Pena Blanca Lake, 1170m, 7 Nov 2010 J. Schaller |  | KU937735 |
| *B.* (*Neobrachinus*) *lateralis* Aggregation B | 2270 | USA: Arizona, Pinal Co. 3 mi NE Winkleman, Gila River, 600m, 17 Mar 2011, coll. J. Schaller | KU937664 | KU937740 |
| *B.* (*Neobrachinus*) *mexicanus* | 2170 | USA: Arizona, Santa Cruz Co., Pajarito Mts., Sycamore Canyon, 1190m, 13 Mar 2010, coll. W.Moore, J. Schaller, R.L. Smith | KU937632 | KU937706 |
| *B.* (*Neobrachinus*) *mexicanus* | 2171 | USA: Arizona, Santa Cruz Co., Pajarito Mts., Sycamore Canyon, 1190m, 13 Mar 2010, coll. W.Moore, J. Schaller, R.L. Smith | KU937633 | KU937707 |
| *B.* (*Neobrachinus*) *mexicanus* | 2177 | USA: Arizona, Santa Cruz Co., Pajarito Mts., Sycamore Canyon, 1190m, 13 Mar 2010, coll. W.Moore, J. Schaller, R.L. Smith | KU937639 | KU937713 |
| *B.* (*Neobrachinus*) *mexicanus* | 2178 | USA: Arizona, Cochise Co., San Pedro River, Charleston Bridge, 1200m, 23 Feb 2010, coll. W.Moore, D. and J. Maddison | KU937640 | KU937714 |
| *B.* (*Neobrachinus*) *mexicanus* | 2205 | USA: Arizona: Yavapai Co.,T18N, R6W sec. 25, Walnut Creek, 8 Jul 2004, coll. R. Delph | KU937646 |  |
| *B.* (*Neobrachinus*) *mexicanus* | 2223 | USA: Arizona, Santa Cruz Co., Pajarito Mts., Sycamore Canyon, 1190m, 13 Mar 2010, coll. W.Moore, J. Schaller, R.L. Smith | KU937653 | KU937725 |
| *B.* (*Neobrachinus*) *mexicanus* | 2224 | USA: Arizona, Santa Cruz Co., Pajarito Mts., Sycamore Canyon, 1190m, 13 Mar 2010, coll. W.Moore, J. Schaller, R.L. Smith | KU937654 | KU937726 |
| *B.* (*Neobrachinus*) *mexicanus* | 2225 | USA: Arizona, Santa Cruz Co., Pajarito Mts., Sycamore Canyon, 1190m, 13 Mar 2010, coll. W.Moore, J. Schaller, R.L. Smith | KU937655 | KU937727 |
| *B.* (*Neobrachinus*) *mexicanus* | 2263 | USA: AZ: Santa Cruz Co. Atascosa Mts. Pena Blanca Lake, 1170m, 7 Nov 2010 J. Schaller |  | KU937737 |
| *B.* (*Neobrachinus*) *mexicanus* | 2269 | USA: Arizona, Pinal Co. 3 mi NE Winkleman, Gila River, 600m, 17 Mar 2011, coll. J. Schaller | KU937663 | KU937739 |
| *B.* (*Neobrachinus*) *mexicanus* | 2276 | USA: Arizona, Maricopa Co. Lower Sycamore Creek, off Hwy, 720m, 28 Mar 2011, coll. J. Schaller | KU937670 | KU937746 |
| *B.* (*Neobrachinus*) *neglectus* | 2316 | USA: SC: Hampton Co., 7km WSW Garnett, Webb Wildlife Mgt. Area, Bluff Lake, 19m, 7 Apr 2006, coll. R. Androw, K. Karns | KU937687 | KU937763 |
| *B.* (*Neobrachinus*) *ovipennis* | 2222 | USA: Vermont, Chittenden Co., Colchester, Delta Park, 30m, 15 Jun 2010, coll. W. Moore | KU937652 | KU937724 |
| *B.* (*Neobrachinus*) *pallidus* | 2299 | USA: CA: Yolo Co. Rd. 57, Cache Creek, 130m | KU937680 | KU937758 |
| *B.* (*Neobrachinus*) *phaeocerus* | 2175 | USA: Arizona, Cochise Co., San Pedro River, Charleston Bridge, 1200m, 23 Feb 2010, coll. W.Moore, D. and J. Maddison | KU937637 | KU937711 |
| *B.* (*Neobrachinus*) *phaeocerus* | 2176 |  | KU937638 | KU937712 |
| *B.* (*Neobrachinus*) *phaeocerus* | 2321 | USA: OK: Washita Co., Rocky, Rocky Lake, 7 Oct 2012, coll. G.F. Hevel | KU937689 | KU937765 |
| *B.* (*Neobrachinus*) *phaeocerus* | 2322 | USA: OK: Washita Co., Rocky, Rocky Lake, 7 Oct 2012, coll. G.F. Hevel | KU937690 | KU937766 |
| *B.* (*Neobrachinus*) *phaeocerus* | 2277 | USA: Arizona, Maricopa Co. Lower Sycamore Creek, 720m, 28 Mar 2011, coll. J. Schaller | KU937671 | KU937747 |
| *B.* (*Neobrachinus*) *quadripennis* | 2336 | USA: FL: Columbia Co. Osceola NF, 40m, 18 Aug 2012, coll. P. Gorring, L. Davis | KU937694 | KU937770 |
| *B.* (*Neobrachinus*) *quadripennis* | 2337 | USA: FL: Columbia Co. Osceola NF, 40m, 18 Aug 2012, coll. P. Gorring, L. Davis | KU937695 | KU937702 |
| *B.* (*Neobrachinus*) *sublaevis* | 2356 | USA: AR: Garland Co., FS Camp Clearfork, 20 Jun 2009, coll. B. Baldwin | KU937700 |  |
| *B.* (*Neobrachinus*) *sp. C* | 2303 | PARAGUAY: Central Department Capiata, 7 Dec 2005, coll. K. Will | KU937684 | KU937760 |
| *B.* (*Neobrachinus*) *sp. E* | 2318 | GUYANA: Region 9, Karanambu Lodge, 14-17 Sep 2012, 100m, coll. D. Brzoska |  | KU937701 |
