# Supplementary material for: Molecular phylogeny, ecology and multispecies aggregation behaviour of bombardier beetles in Arizona
Source: PLoS One. 2018 Oct 31;13(10):e0205192. doi: 10.1371/journal.pone.0205192 (PMC6209175; doi:10.1371/journal.pone.0205192)
Supplement: S2 Table — Parentheses show the expected number of individuals per species in each aggregation if all individuals settle at random with respect to the identity and relative abundance of species collected at Site 2. P value is the probability of finding the observed number of species in each aggregation based on a randomization test. (DOCX) [file pone.0205192.s002.docx]

|  | *Brachinus elongatulus* | *Brachinus mexicanus* | *Brachinus hirsutus* | *Brachinus imporcitis* | *Brachinus favicollis* | *Brachinus gebhardis* | P |
| --- | --- | --- | --- | --- | --- | --- | --- |
| Aggregation 32 | 0 (0.2) | 8 (6) | 21 (29) | 12 (5) | 0 (1) | 0 (0.2) | 0.20 |
| Aggregation 33 | 0 (0.1) | 1 (2) | 12 (10) | 1 (2) | 0 (0.4) | 0 (0.1) | 0.44 |
| Aggregation 34 | 0 (0.1) | 1 (2) | 15 (11) | 0 (2) | 0 (0.4) | 0 (0.1) | 0.05^§^ |
| Aggregation 35 | 0 (0.04) | 2 (1) | 4 (5) | 1 (1) | 0 (0.2) | 0 (0.04) | 0.30 |
| Aggregation 36 | 1 (0.1) | 8 (3) | 10 (17) | 4 (3) | 1 (0.6) | 0 (0.3) | 0.047* |
| Aggregation 37 | 0 (0.09) | 1 (2) | 15 (11) | 0 (2) | 0 (0.4) | 0 (0.09) | 0.051 |
| Aggregation 38 | 0 (0.1) | 1 (3) | 14 (13) | 4 (2) | 0 (0.5) | 0 (0.1) | 0.41 |
| Aggregation 39 | 0 (0.04) | 0 (1) | 7 (5) | 0 (1) | 0 (0.2) | 0 (0.04) | 0.076 |
| Aggregation 40 | 0 (0.05) | 0 (1) | 10 (7) | 0 (1) | 0 (0.3) | 0 (0.05) | 0.028^§^ |
| Aggregation 41 | 0 (0.04) | 1 (1) | 6 (5) | 0 (1) | 0 (0.2) | 0 (0.04) | 0.20 |
| Aggregation 42 | 0 (0.03) | 0 (0.7) | 5 (3) | 0 (0.6) | 0 (0.1) | 0 (0.03) | 0.17 |
| Aggregation 43 | 0 (0.03) | 2 (0.8) | 4 (4) | 0 (0.8) | 0 (0.2) | 0 (0.03) | 0.22 |
| Aggregation 44 | 0 (0.01) | 0 (0.4) | 0 (2) | 0 (0.4) | 3 (0.1) | 0 (0.01) | < 0.0001^¶^ |
| Aggregation 45 | 0 (0.01) | 0 (0.3) | 1 (1) | 0 (0.3) | 1 (0.05) | 0 (0.01) | 0.037* |
| Aggregation 46 | 0 (0.01) | 0 (0.4) | 2 (2) | 0 (0.4) | 0 (0.1) | 1 (0.01) | 0.008* |
| Aggregation 47 | 0 (0.01) | 0 (0.3) | 1 (1) | 1 (0.3) | 0 (0.05) | 0 (0.01) | 0.18 |

§ Number of species in aggregation was lower than expected assuming individuals settle at random

*Number of species in aggregation was higher than expected assuming individuals settle at random

¶ Aggregation was not random because it consisted solely of three individuals from a relatively rare species.
