# Supplementary material for: Molecular phylogeny, ecology and multispecies aggregation behaviour of bombardier beetles in Arizona
Source: PLoS One. 2018 Oct 31;13(10):e0205192. doi: 10.1371/journal.pone.0205192 (PMC6209175; doi:10.1371/journal.pone.0205192)
Supplement: S3 Table — Parentheses show the expected number of individuals per species in each aggregation if all individuals settle at random with respect to the identity and relative abundance of species collected at Site 3. P value is the probability of finding the observed number of species in each aggregation based on a randomization test. (DOCX) [file pone.0205192.s003.docx]

|  | *Brachinus elongatulus* | *Brachinus mexicanus* | *Brachinus hirsutus* | *Brachinus favicollis* | *Brachinus costipennis* | *Brachinus lateralis* | P |
| --- | --- | --- | --- | --- | --- | --- | --- |
| Aggregation 22 | 1 (0.7) | 10 (12) | 1 (0.7) | 1 (0.2) | 0 (0.1) | 1 (0.1) | 0.003^*^ |
| Aggregation 23 | 1 (1) | 21 (20) | 1 (1) | 0 (0.3) | 0 (0.2) | 0 (0.2) | 0.25 |
| Aggregation 24 | 1 (0.8) | 14 (14) | 1 (0.8) | 0 (0.2) | 0 (0.1) | 0 (0.1) | 0.20 |
| Aggregation 25 | 1 (1) | 26 (24) | 0 (1) | 1 (0.4) | 0 (0.2) | 0 (0.2) | 0.044^¶^ |
| Aggregation 26 | 0 (0.6) | 11 (11) | 2 (0.6) | 0 (0.2) | 0 (0.09) | 0 (0.09) | 0.18 |
| Aggregation 27 | 2 (0.8) | 13 (14) | 1 (0.8) | 0 (0.2) | 0 (0.1) | 0 (0.1) | 0.20 |
| Aggregation 28 | 1 (0.7) | 13 (13) | 0 (0.7) | 0 (0.2) | 1 (0.1) | 0 (0.1) | 0.019^*^ |
| Aggregation 29 | 0 (0.3) | 6 (5) | 0 (0.3) | 0 (0.08) | 0 (0.04) | 0 (0.04) | 0.44 |
| Aggregation 30 | 0 (0.2) | 5 (4) | 0 (0.2) | 0 (0.07) | 0 (0.04) | 0 (0.04) | 0.50 |
| Aggregation 31 | 0 (0.1) | 2 (3) | 1 (0.1) | 0 (0.04) | 0 (0.02) | 0 (0.02) | 0.12 |

*Number of species in aggregation was higher than expected assuming individuals settle at random

¶ Aggregation was not random because it did not contain individuals of a relatively common species but contained one individual of a relatively rare species
