# Supplementary material for: Molecular phylogeny, ecology and multispecies aggregation behaviour of bombardier beetles in Arizona
Source: PLoS One. 2018 Oct 31;13(10):e0205192. doi: 10.1371/journal.pone.0205192 (PMC6209175; doi:10.1371/journal.pone.0205192)
Supplement: S4 Table — Parentheses show the expected number of individuals per species in each aggregation if all individuals settle at random with respect to the identity and relative abundance of species collected at Site 6. P value is the probability of finding the observed number of species in each aggregation based on a randomization test. (DOCX) [file pone.0205192.s004.docx]

|  | *Brachinus elongatulus* | *Brachinus mexicanus* | *Brachinus hirsutus* | P |
| --- | --- | --- | --- | --- |
| Aggregation 1 | 6 (5) | 0 (0.5) | 0 (0.02) | 0.55 |
| Aggregation 2 | 12 (14) | 3 (1) | 0 (0.06) | 0.71 |
| Aggregation 3 | 22 (22) | 2 (2) | 0 (0.1) | 0.81 |
| Aggregation 4 | 8 (7) | 0 (0.7) | 0 (0.03) | 0.45 |
| Aggregation 5 | 5 (5) | 0 (0.4) | 0 (0.02) | 0.62 |
| Aggregation 6 | 73 (67) | 1 (7) | 0 (0.3) | 0.74 |
| Aggregation 7 | 32 (29) | 0 (3) | 0 (0.1) | 0.041^§^ |
| Aggregation 8 | 20 (24) | 6 (2) | 0 (0.1) | 0.83 |
| Aggregation 9 | 21 (22) | 2 (2) | 1 (0.1) | 0.084 |
| Aggregation 10 | 5 (7) | 3 (0.7) | 0 (0.03) | 0.52 |
| Aggregation 11 | 0 (5) | 5 (0.4) | 0 (0.02) | <0.0001^¶^ |
| Aggregation 12 | 17 (15) | 0 (2) | 0 (0.07) | 0.19 |

§ Number of species in aggregation was lower than expected assuming individuals settle at random

¶ Aggregation was not random because it did not contain individuals of a relatively common species but contained five individuals of a relatively rare species.
