# Supplementary material for: Molecular phylogeny, ecology and multispecies aggregation behaviour of bombardier beetles in Arizona
Source: PLoS One. 2018 Oct 31;13(10):e0205192. doi: 10.1371/journal.pone.0205192 (PMC6209175; doi:10.1371/journal.pone.0205192)
Supplement: S5 Table — Parentheses show the expected number of individuals per species in each aggregation if all individuals settle at random with respect to the identity and relative abundance of species collected at Site 7. P value is the probability of finding the observed number of species in each aggregation based on a randomization test. (DOCX) [file pone.0205192.s005.docx]

|  | *Brachinus elongatulus* | *Brachinus mexicanus* | *Brachinus hirsutus* | *Brachinus gebhardis* | *Brachinus lateralis* | P |
| --- | --- | --- | --- | --- | --- | --- |
| Aggregation 13 | 90 (86) | 0 (2) | 0 (0.9) | 0 (0.2) | 0 (0.5) | 0.027^§^ |
| Aggregation 14 | 57 (68) | 8 (2) | 3 (0.7) | 0 (0.1) | 3 (0.4) | 0.001^*^ |
| Aggregation 15 | 33 (33) | 1 (0.7) | 0 (0.3) | 0 (0.07) | 0 (0.2) | 0.29 |
| Aggregation 16 | 10 (11) | 1 (0.2) | 0 (0.1) | 0 (0.02) | 0 (0.7) | 0.18 |
| Aggregation 17 | 37 (35) | 0 (0.8) | 0 (0.4) | 0 (0.07) | 0 (0.2) | 0.22 |
| Aggregation 18 | 9 (10) | 0 (0.2) | 0 (0.1) | 1 (0.02) | 0 (0.06) | 0.014^*^ |
| Aggregation 19 | 3 (5) | 0 (0.1) | 2 (0.05) | 0 (0.01) | 0 (0.03) | 0.044^*^ |
| Aggregation 20 | 2 (2) | 0 (0.7) | 0 (0.01) | 0 (0.004) | 0 (0.01) | 0.93 |
| Aggregation 21 | 243 (234) | 1 (5) | 0 (2) | 0 (0.5) | 0 (1) | 0.012^§^ |

§ Number of species in aggregation was lower than expected assuming individuals settle at random

* Number of species in aggregation was higher than expected assuming individuals settle at random
